# “Unassisted by Education, and Unfettered by the Rules of Art”

**DOI:** 10.3201/eid2906.AC2906

**Published:** 2023-06

**Authors:** Byron Breedlove

**Affiliations:** Centers for Disease Control and Prevention, Atlanta, Georgia, USA

**Keywords:** art science connection, emerging infectious diseases, art and medicine, about the cover, public health, John Williamson, Recovering Smallpox Patient, c.1770, “unassisted by education, and unfettered by the rules of art,” smallpox, monkeypox virus, mpox, poxviruses, viruses, variola virus, Johnnie Notions, inoculation, vaccination, Edward Jenner, wigs, wig-stretching block, zoonoses

**Figure:**
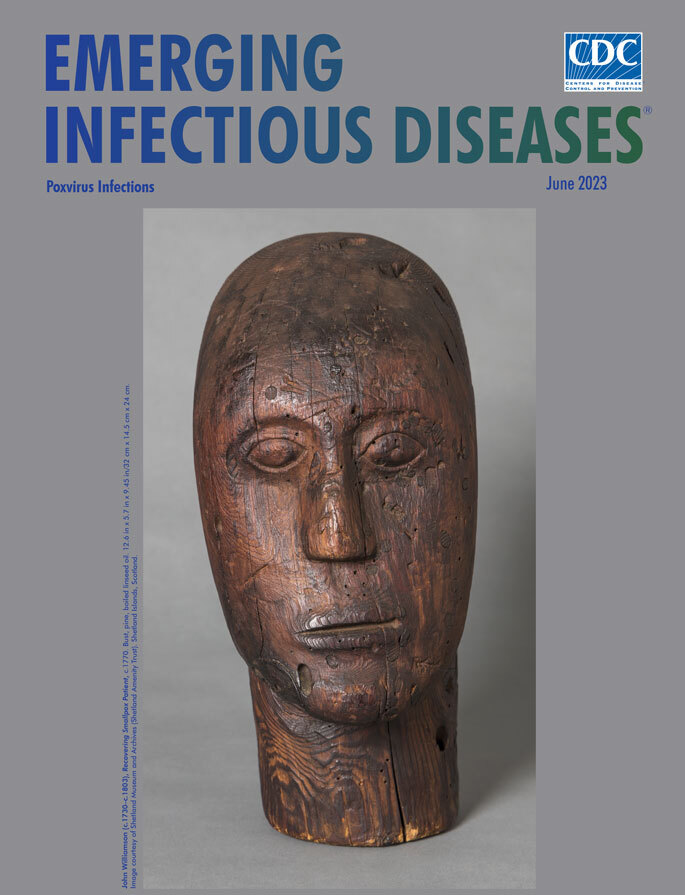
**John Williamson (c.1730–c.1803), *Recovering Smallpox Patient*, c.1770.** Bust, pine, boiled linseed oil. 12.6 in x 5.7 in x 9.45 in/32 cm x 14.5 cm x 24 cm. Shetland Museum and Archives. Shetland Islands, Scotland, UK.

The wig-stretching block appearing on this month’s cover is thought to be the only surviving work from John Williamson, who lived on the Shetland Islands of Scotland. Williamson, perhaps better known by his nickname “Johnnie Notions” (sometimes spelled Johnie), proved to be skilled at many trades. *The Statistical Accounts of Scotland 1791−1845* describes him as "a singular instance of an uncommon variety of talents, being a tailor, a joiner, a clock and watch-mender, a blacksmith, and a physician." He was also, at various times in his life, a farmer, fisherman, and weaver. A self-taught physician, Williamson developed and administered smallpox inoculations for an estimated 3,000 patients during the late 18th century, primarily during the decade before Edward Jenner developed his smallpox vaccine in 1796. The procedure Williamson used, known as variolation, had been used for thousands of years, and it involves taking live virus from a smallpox patient and “inoculating” that virus onto the skin of a recipient who did not have the disease. 

Ian D. Conacher, a physician and researcher, and Brian Smith, an archivist and author with the Shetland Archives, have each authored detailed accounts of Williamson’s life and his work as an inoculator. Both authors provide historical context for how smallpox was introduced from the mainland of Scotland to Shetland and the devastating consequences resulting from a series of smallpox outbreaks, curiously occurring in 20-year intervals (1700, 1720, 1740, and 1760). Both also draw from *The Statistical Accounts of Scotland 1791−1845,* which provides this account of Williamson’s work:

“Williamson was a local pioneer in the use of vaccines against smallpox. Unassisted by education, and unfettered by the rules of art. He is careful in providing the best matter and keeps it a long time before he puts it to use - sometimes 7 or 8 years. And, in order to lessen its virulence, he first dries it in peat smoke, and then puts it under the ground covered in camphor....by a small knife, made by his own hand, he gently raises a very little of the outer skin of the arm ....then puts in a very small quantity of the matter....The only plaister he uses, for healing the wound, is a bit of cabbage leaf.”

Conacher noted that there is no evidence that Williamson caused any cases of smallpox or deaths among the people he inoculated. However, it is difficult to determine whether his techniques to reduce virulence of the virus were always successful. Variolation was inherently risky, in that, for some, the process could result in a full-blown case of smallpox. Regardless of the resulting severity, the individual recipient could then transmit the virulent smallpox virus to others.

 According to the Shetland Museum and Archives, Williamson created the wig block for the Cheyne family, lairds at Tangwick Haa, Northmavine, Shetland, and “The face is modelled on an old man suffering from smallpox, the wood chosen because it was naturally marked with similar scarring.” The archivist Smith wrote that “There is a strong tradition in Shetland that ‘Johnie Notions’ used as his model for this stretcher an old man from Hillswick whose face was marked with smallpox.” A careful observer might conclude that, given the few pockmarks on the face, the man had experienced a relatively mild case of smallpox. 

During that time in Europe, wigs were often favored by the wealthy and aristocratic classes but not simply to signal wealth and social status. Syphilis, which was widespread across Europe, caused hair loss and skin sores, so wigs helped disguise those symptoms. Blocks such as the one Williamson created from a piece of pine were essential for preening, shaping, and maintaining wigs. 

During the last years of Williamson’s life, Jenner developed the first vaccine against smallpox and envisioned the elimination of smallpox. In his 1801 treatise *On the Origin of the Vaccine Inoculation*, Jenner wrote, “the annihilation of the smallpox, the most dreadful scourge of the human species, must be the final result of this practice.” 

A good measure of control was achieved in many developed countries, largely via mass vaccination campaigns. However, because of poor health infrastructures in most developing countries, mass campaigns were not very successful, and smallpox circulation continued into the 20th century. In the 1960s, the deployment of an approach known as surveillance and containment—in which cases of smallpox were identified and traced and all those exposed and nearby were vaccinated—proved successful. Consequently, in 1980, the World Health Organization declared smallpox to be eradicated. 

Although the smallpox virus (variola virus), the best known of the poxviruses affecting humans, no longer exists naturally, other poxviruses, including monkeypox virus, orf virus, molluscum contagiosum, yatapoxviruses, and parapoxviruses, can infect humans or other animals. The recent epidemic of mpox highlights the importance of continued vigilance, response, prevention, and innovation in public health efforts to control and treat diseases caused by poxviruses.
